# Choroidal structural changes correlate with severity of diabetic retinopathy in diabetes mellitus

**DOI:** 10.1186/s12886-019-1189-8

**Published:** 2019-08-16

**Authors:** Hui Wang, Yong Tao

**Affiliations:** Department of Ophthalmology, Beijing Chao-yang Hospital, Capital Medical University, Beijing, 100020 People’s Republic of China

**Keywords:** Diabetic retinopathy, Choroidal vascular density parameter, Choroid, Macular

## Abstract

**Background:**

This study aims to investigate the choroidal thickness and choroidal vascular density parameters and their correlation with severity of diabetic retinopathy (DR) in diabetes mellitus (DM) patients.

**Methods:**

An observational cross-sectional study was conducted of 104 eyes, which were divided into 4 groups: Healthy controls (*n* = 38), DM with no DR eyes (*n* = 22), panretinal photocoagulation-untreated non-proliferative DR eyes (PRP-untreated NPDR eyes) (*n* = 24), PRP-untreated proliferative DR eyes (PRP-untreated PDR eyes) (*n* = 20). Optical coherence tomography (OCT) was performed. The total choroidal area (TCA), stromal area (SA), the luminal areas (LA) and the ratio of the luminal to choroidal area (L/C ratio) were compared. The choroidal parameters were also compared between PRP untreated and PRP-treated DR eyes.

**Results:**

The L/C ratio values were 0.68 ± 0.06 in controls and 0.63 ± 0.04 in DM eyes (*P* < 0.001). But there were no statistically significant differences in retinal nerve fiber layer (RNFL) thickness, retinal thickness and subfoveal choroidal thickness (SCT) measurements between the two groups (*P* = 0.407, *P* = 0.654 and *P* = 0.849; respectively). The vessel density values were significantly different in DM with no DR eyes, PRP-untreated NPDR eyes and PRP-untreated PDR eyes (*P* < 0.001 for SCT, TCA and SA). The L/C ratio values in the three groups were significant different (*P* = 0.019). There was no significant difference in SCT, TCA, LA, SA and the L/C ratio between PRP-untreated and PRP-treated DR eyes.

**Conclusion:**

Eyes of patients with DM showed the L/C ratio decreased compared with normal controls. The SCT increased, but L/C ratio significantly decreased with severity of DR eyes compared with DM and normal eyes. Changes in the L/C ratio may predict DR development before they are otherwise evident clinically. Choroidal blood flow deficit can be an early pathologic change in DR.

**Electronic supplementary material:**

The online version of this article (10.1186/s12886-019-1189-8) contains supplementary material, which is available to authorized users.

## Background

The chronic hyperglycemia of diabetes mellitus (DM) can cause microvascular abnormalities [1] and complications of eyes [2]. It affects the retinal circulation and the choroidal vasculature. Diabetic retinopathy (DR) is one of the most severe complications of DM which can cause permanent visual impairment and affect the quality of life [3, 4]. DR is gradually occurring with the development of DM. But due to the limitations of clinical diagnostic techniques, the pathological changes of DR have already occurred and has a certain effect on the patient’s vision when diagnosed. It is essential to improve the early diagnosis rate of DR.

The capillary closure resulting in non-perfusion of retinal capillaries is the most important pathologies in DR. [5] Hidayat et al. [6] reported the choroidal abnormal performance included capillary drop-out, luminal narrowing of capillaries and choroidal neovascularization. Choroidal thickness (CT) is a parameter to evaluate abnormalities in choroidal vasculature. There is increasing interest in development of quantitative methods to assess choroidal structural characteristics and their associations with ocular diseases. Advancement in technology has provided further insight into both qualitative and quantitative measurements of choroidal vasculature and other volumetric data in choroidal pathology. Many recent studies focus on using CT as an indicator of retinal and choroidal blood flow [7–10]. But various variables can affect CT and retinal vessels. So, there is a need to explore more robust and stable marker for the assessment of retinal and choroidal vascular structural characteristics.

After the image segmentation technique proposed by Sonoda et al. [11, 12], Agrawal et al. [13] proposed a new parameter-choroidal vascularity index (CVI) to assess vascular structure through enhanced depth imaging optic coherence tomography (EDI-OCT) images. After binarization of the OCT images, the total choroidal area (TCA), the stromal area (SA) as well as vascular luminal areas (LA) are identified and measured. With growing evidence, CVI is emerging as a potentially more robust marker and a complimentary tool for choroidal vascularity in various ocular diseases. CVI can indirectly measure choroidal vascularity quantitatively, overcoming the limitation of using CT alone [14]. In the study of Kim [14], they assessed choroidal changes in diabetic patients by measuring CVI and CT in conjunction with DR stage. But they did not assessed the other indicators, such as TCA, SA and LA. In this study, we aimed to determine the difference in the choroidal vasculature in patients with DM, NPDR, PDR and in healthy controls by measuring choroidal vascular density parameters.

## Methods

### Study population

This was an observational cross-sectional study. To determine the difference in the choroidal vasculature in patients with DM, non-proliferative DR (NPDR), proliferative DR (PDR) and in healthy controls. 104 eyes were included in this study. Eyes were divided into 4 groups: Healthy controls (*n* = 38), DM with no DR eyes (*n* = 22), panretinal photocoagulation-untreated NPDR eyes (PRP-untreated NPDR eyes) (*n* = 24), PRP-untreated PDR eyes (PRP-untreated PDR eyes) (*n* = 20). The last 3 groups were also called the DM groups (*n* = 66).

After the patients were included in the study, 40 patients (20 NPDR patients and 20 PDR patients) underwent PRP treatment, but 3 patients failed to return in time. To investigate the difference before and after PRP treatment, the choroidal parameters of 24 PRP-untreated NPDR, 17 PRP-treated NPDR eyes, 20 PRP-untreated and PRP-treated eyes were also assessed in this study.

The severity of the diabetic eye disease was graded according to the Early Treatment Diabetic Retinopathy Study (ETDRS). The study was conducted with the approval from the Ethics Committee of Beijing Chaoyang Hospital, the Third Clinical Medical College of Capital Medical University. All procedures performed in studies involving human participants were in accordance with the 2013 Helsinki declaration. Written informed consent was obtained from the subjects after explanation about any potential risks involved with the study.

The inclusion criteria were (1) spherical equivalent refractive error < 6.00 diopter or axial length no more than 26 mm; (2) obscuration of choroidal images by existence of significant media opacity or thick subfoveal hemorrhage; (3) bilateral pathological myopia; (4) previous vitrectomy, intraocular surgery (including cataract surgery) in the study eye within 2 years.

### Clinical examination

All patients underwent standardized measurement of best-corrected visual acuity (BCVA), intraocular pressure (IOP), slit-lamp biomicroscopy, dilated fundal examination, fluorescence angiography (FA) with a confocal scanning laser system (HRA Spectralis; Heidelberg Engineering, Germany), and the EDI-OCT scan was performed using the Heidelberg Spectralis (Version 5.3.2.0; Heidelberg Engineering, Heidelberg, Germany). Snellen visual acuities were converted to logMAR equivalent for statistical analysis.

### Measurement of retinal and choroidal thickness

EDI-OCT scans of the macula were performed for all eyes using spectral-domain OCT (Spectralis, Heidelberg Engineering, Heidelberg, Germany) before and after PRP treatment. 38 healthy eyes, 22 DM with no DR eyes, 24 PRP-untreated NPDR eyes, 20 PRP-untreated PDR eyes before fundus laser treatment and 37 eyes (17 NPDR and 20 PDR eyes) after PRP treatment performed OCT examinations.

**Fig. 1:**
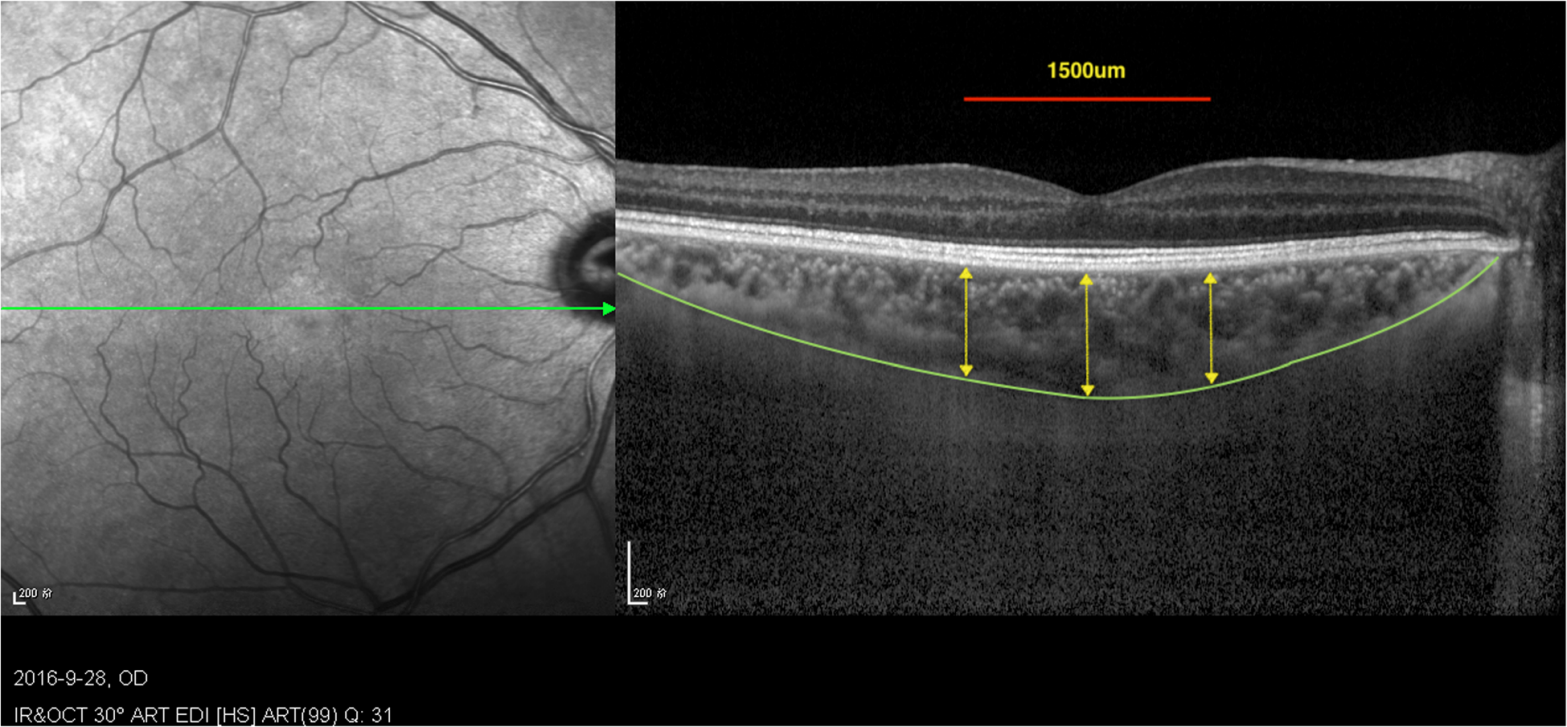
SCT measurements. For SCT measurements, we manually moved the automatically segmented internal limiting membrane line to the choroidoscleral junction. Once we changed the automatically segmented line and set a vertical line perpendicular to the center of the fovea, the SCT was automatically calculated

The retinal thickness, choroidal thickness and RNFL thickness were measured using the in-built callipers tool at three points (subfoveal, 0.5 mm temporal and nasal to the fovea) (Fig. 1). Only 0.5 mm nasal to the fovea retinal thickness and RNFL thickness were included in statistical analysis.

### Image Binarization and choroidal vascular density parameters calculation

**Fig. 2:**
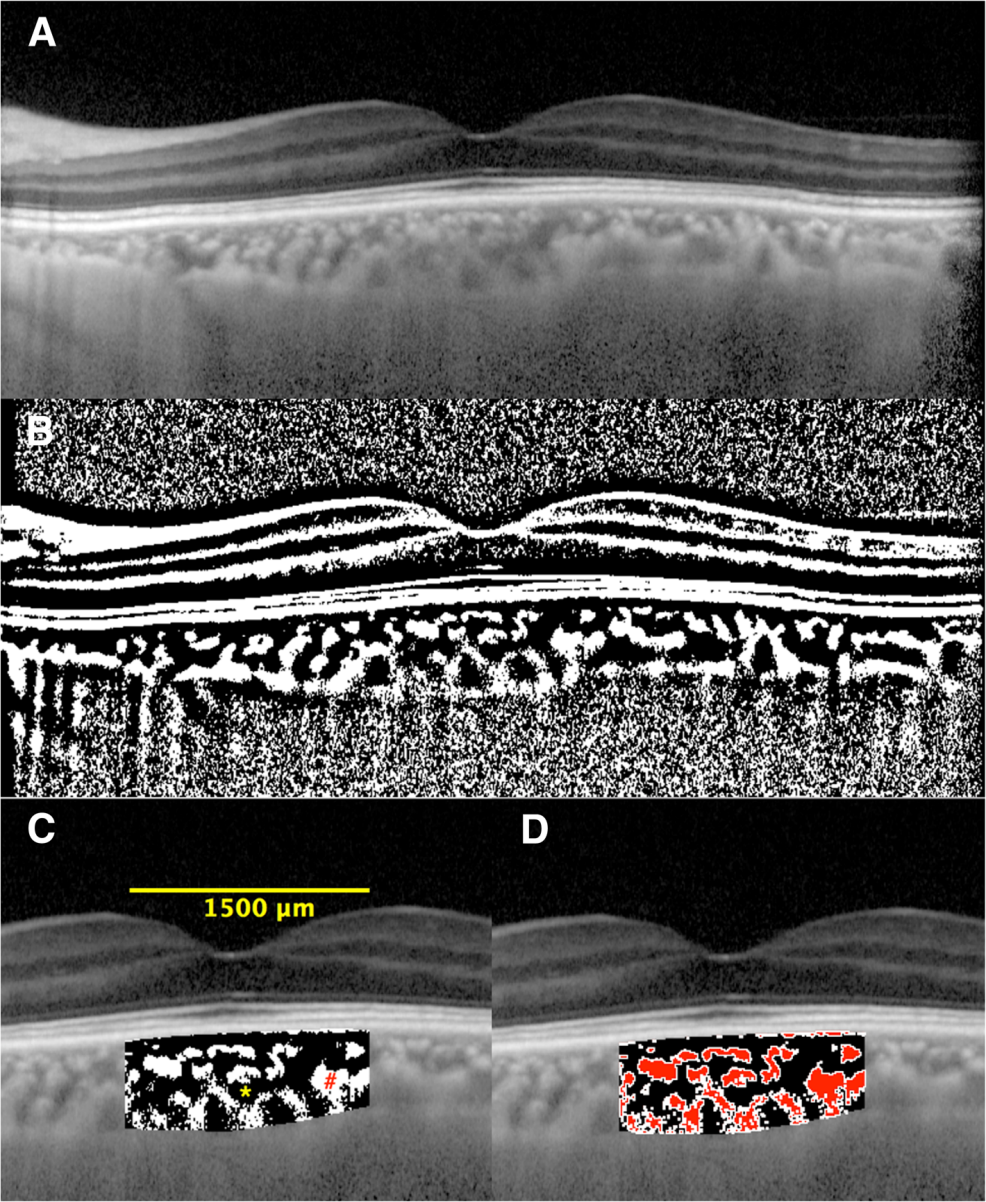
Enhanced depth imaging (EDI)-OCT image and converted binary image of a normal healthy eye. An EDI-OCT image of a healthy eye (**a**) was converted to a binary image (**b**) using the ImageJ software. The luminal area (dark area, asterisk) and the interstitial area (cross) are seen. The rectangle surrounded by a red line was excised, and the dark areas were traced by the Niblack method ([**c**], brown area). The binarized image (**b**) and the margin of traced area (**c**) were merged, and the merged image shows that the traced areas coincide with the dark choroidal areas of the OCT image (**d**)

One central scan passing through the fovea was selected for binarization using the protocol described by Sonoda et al. [11] Raster scans through the fovea in B-scan OCT were binarized using the Niblack autolocal threshold tool. The image binarization was done using public domain software, Image J. Binarization of the subfoveal choroidal area in the OCT image was done by a modified Niblack method. (Fig. 2). After binarization of the OCT images, the TCA, SA as well as LA are identified and measured. The TCA was calculated by multiplying the standard width of 1500 um (750 um on nasal and temporal side of the fovea) by the subfoveal choroidal thickness (SCT). The ratio of the luminal to choroidal area (L/C ratio) was calculated as the ratio of LA over the TCA.

### Statistical analysis

The Kolmogorov–Smirnov test was used to identify the normality of distribution. Descriptive statistics were calculated as the mean and standard deviation for normally distributed variables and median, first quartile, and third quartile for nonnormally distributed variables. The categorical data were analyzed using the Fisher’s exact test. The Independent t-test, one-way analysis of variance (ANOVA) test for normal distributions and Kruskal-Wallis tests for nonnormal distributions were used to compare other parameters between groups. The Tamhane’s T2 test was performed to adjust for multiple comparisons between groups within each analysis.

All reported *P* values were two sided. *P* < 0.05 was considered statistically significant. Statistical analysis was performed using the SPSS software version 21 (SPSS, Inc., IL, USA).

## Results

### Demographic and clinical characteristics

The demographic, ocular, and systemic characteristics of the subjects are shown in Table 1. The study population consisted of 38 healthy controls (mean age, 63.42 ± 7.24 years; male/female, 21/17), 22 DM with no DR patients (mean age, 61.77 ± 7.63 years; male/female, 12/10), 24 PRP-untreated NPDR patients (mean age, 61.83 ± 8.62 years; male/female, 12/12), and 20 PRP-untreated PDR patients (mean age, 63.55 ± 6.39 years; male/female, 9/11), respectively.

**Table 1:** Epidemiologic Characteristics and Ophthalmologic Clinical Presentation of Patients

	Control	DM with no DR	PRP-untreated NPDR	PRP-untreated PDR	*P* value
Number of eyes (*n*)	38	22	24	20	
Age (year), mean ± SD	63.42 ± 7.24	61.77 ± 7.63	61.83 ± 8.62	63.55 ± 6.39	*P* = 0.739^*^
Gender (male/female)	21/17	12/10	12/12	9/11	*P* = 0.457^#^
Years with diabetes (year), mean ± SD	–	5.23 ± 1.31	9.85 ± 2.32	11.68 ± 2.63	P < 0.001^*,†^
BMI (kg/m^2^), mean ± SD	24.38 ± 2.53	26.06 ± 2.53	24.64 ± 3.94	23.77 ± 3.22	*P* = 0.090^*^
HbA1c (%), mean ± SD	–	7.54 ± 0.76	7.96 ± 0.84	8.51 ± 0.96	P = 0.002^*^
Fasting blood glucose (mmol/L), mean ± SD	–	7.37 ± 0.73	7.53 ± 0.91	7.85 ± 1.01	*P* = 0.218^*^
Systolic BP (mmHg), mean ± SD	124.76 ± 3.78	122.36 ± 5.49	123.54 ± 4.36	124.75 ± 4.96	*P* = 0.202^*^
Diastolic BP (mmHg), mean ± SD	80.16 ± 5.81	79.64 ± 5.21	78.96 ± 5.38	79.55 ± 4.83	*P* = 0.865^*^
Diabetic nephropathy (*n*)	0	0	3	5	–
IOP (mmHg), mean ± SD	15.12 ± 1.48	15.14 ± 2.71	15.05 ± 3.23	13.95 ± 4.02	*P* = 0.434^*^
Axial length (mm), mean ± SD	24.10 ± 0.64	23.86 ± 0.67	24.27 ± 0.55	24.01 ± 0.59	*P* = 0.149^*^
Treatment with insulin, *n* (%)	0	19 (86.3)	23 (95.8)	20 (100)	–

SD, standard deviation; BMI, body mass index; BP, blood pressure; DM, diabetes mellitus; DR, diabetic retinopathy; PDR, proliferative diabetic retinopathy; NPDR, non-proliferative diabetic retinopathy; IOP, intraocular pressure

*: Statistical significance tested by ANOVA for normal distributions and Kruskal–Wallis tests for nonnormal distributions; all comparisons were corrected with the post hoc test

#: *P* values were calculated using the Fisher’s exact test

†: Significant difference between mean values of DM with no DR, PRP-untreated NPDR, PRP-untreated PDR eyes

Compared with both DM with no DR patients and PRP-untreated NPDR patients, PRP-untreated PDR patients had significantly lower mean self-reported history of diabetes and HbA1c (*P* < 0.001, *P* = 0.002; respectively), and they had also lower BCVA compared with the DM with no DR and PRP-untreated NPDR patients (P < 0.001). There were no statistically significant differences between mean BMI, fasting blood glucose, systolic BP, diastolic BP, IOP and axial length among groups. 62 patients received insulin treatment (19 in DM with no DR eyes, 23 in PRP-untreated NPDR eyes and 20 in PRP-untreated PDR eyes) in all 66 DM patients. (Table 1).

### OCT parameters between healthy controls and diabetes group

**Fig. 3:**
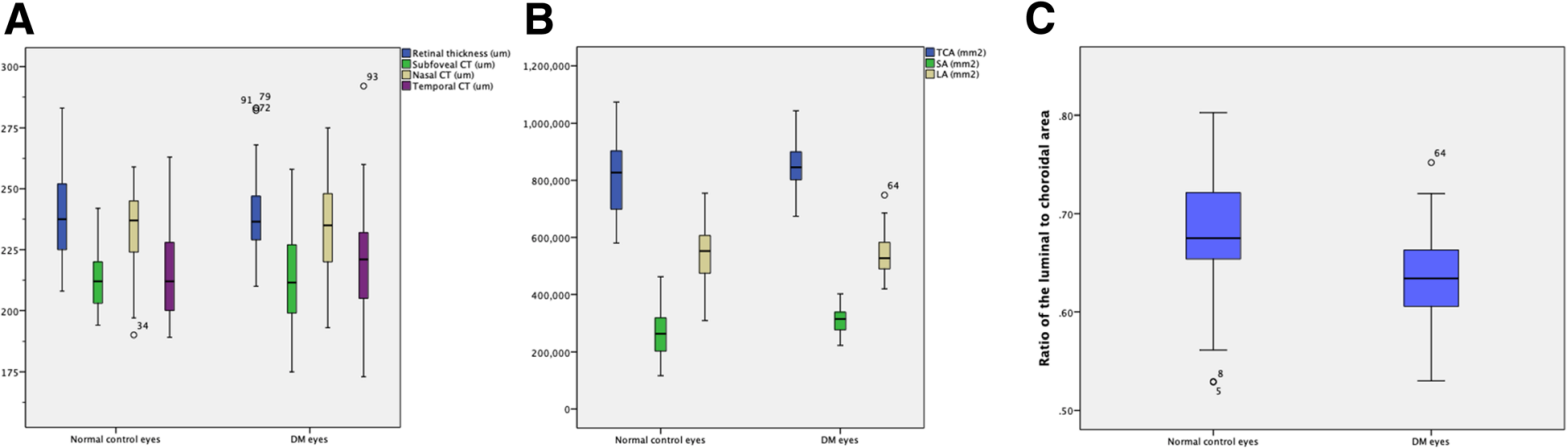
OCT parameters between healthy controls and diabetes group. (**a**) Retinal thickness and SCT measurements between the two groups; (**b**) TCA, SA as well as LA between the two groups; (**c**) The L/C ratio between the two groups

The DM group included the DM with no DR patients, the PRP-untreated NPDR patients and the PRP-untreated PDR patients (*n* = 66). EDI-OCT scans of 38 healthy controls and 66 eyes of patients with DM were analyzed. Independent t-test showed that the L/C ratio values were significantly different between the two groups (*P* < 0.001). But there were no statistically significant differences in RNFL thickness, retinal thickness and SCT between the two groups (*P* = 0.407, *P* = 0.654 and *P* = 0.849; respectively).(Table 2)(Fig. 3).

**Table 2:** OCT parameters between healthy controls and diabetes group

	Control	DM	*P* value
Number of eyes (n)	38	66	
RNFL thickness (um), median (IQR)	11 (10,12.25)	11 (10,13)	P = 0.407^#^
Retinal thickness (um), mean ± SD	240.58 ± 20.64	238.85 ± 15.33	P = 0.654^*^
CT (um), mean ± SD			
Subfoveal	212.63 ± 11.99	213.21 ± 19.02	P = 0.849^*^
Nasal	233.03 ± 16.69	234.05 ± 19.57	*P* = 0.788^*^
Temporal	216.95 ± 19.32	219.71 ± 20.61	*P* = 0.502^*^
Choroid vascular parameters			
TCA in mm^2^, mean ± SD	0.81 ± 0.14	0.85 ± 0.08	*P* = 0.086^*^
LA in mm^2^, mean ± SD	0.54 ± 0.09	0.54 ± 0.07	*P* = 0.825^*^
SA in mm^2^, mean ± SD	0.26 ± 0.07	0.31 ± 0.04	P < 0.001^*,†^
L/C ratio	0.68 ± 0.06	0.63 ± 0.04	P < 0.001^*,†^

OCT, optical coherence tomography; IQR, interquartile range; SD, standard deviation; CT, choroidal thickness; TCA, total choroidal area; LA, luminal area; SA, stromal area; L/C ratio, the ratio of the luminal to choroidal area

*: *P* values were calculated using the Independent t-test

#: *P* values were calculated using the Mann-Whitney U test

†: Significant difference between mean values of control eyes and DM eyes

### Choroidal parameters between DM groups

**Fig. 4:**
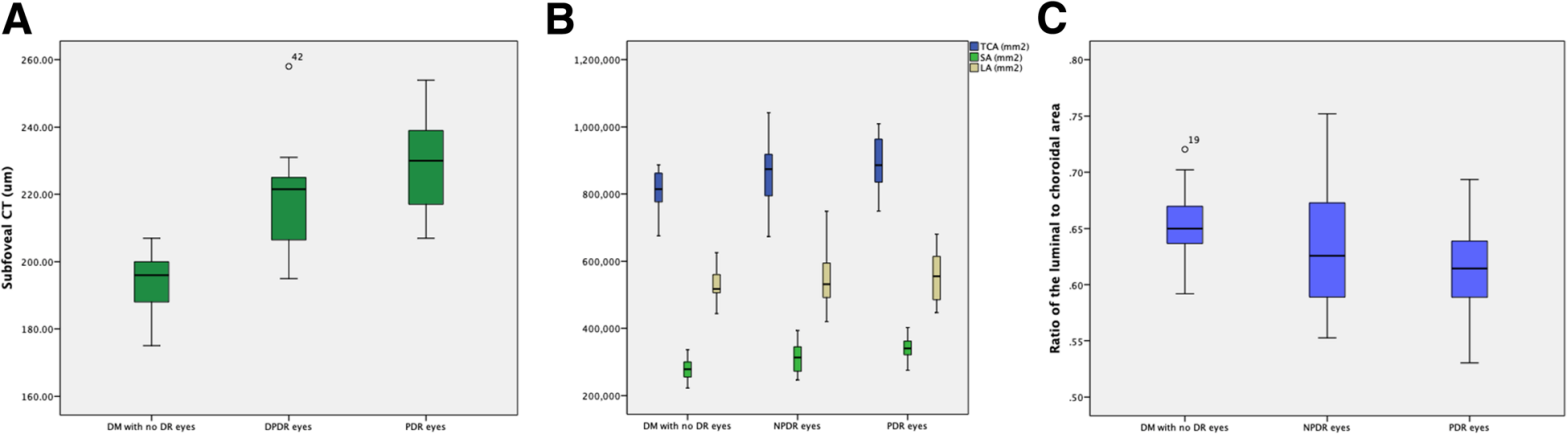
OCT parameters in DM with no DR eyes, PRP-untreated NPDR eyes and PRP-untreated PDR eyes. (**a**) SCT between the three groups; (**b**) TCA, SA as well as LA among the three groups; (**c**) The L/C ratio among the three groups

Age-adjusted 1-way ANOVAs showed that the vessel density values were significantly different among the three groups (*P* < 0.001 for both SCT, TCA and SA). The SCT values were significantly lower in DM with no DR eyes (194.18 ± 5.68 um), followed by PRP-untreated NPDR eyes (217.29 ± 14.07 um) and PRP-untreated PDR eyes (229.25 ± 13.89um); all three pairwise comparisons were statistically significant (Tukey-Kramer HSD, *P* < 0.05 for all comparisons). For TCA and SA, the pairwise comparisons showed that DM with no DR eyes (0.81 ± 0.06 mm^2^; 0.28 ± 0.03 mm^2^) were significantly lower compared with PRP-untreated NPDR eyes, PRP-untreated PDR eyes (0.86 ± 0.09 mm^2^; 0.31 ± 0.05 mm^2^) and healthy eyes (0.90 ± 0.08 mm^2^; 0.34 ± 0.03 mm^2^) (Tukey-Kramer HSD, *P* < 0.001 for both). (Table 3) (Fig. 4).

**Table 3:** Choroidal parameters of all the patients

	No DR eyes	PRP-untreated NPDR eyes	PRP-untreated PDR eyes	*P* value
Number of eyes (n)	22	24	20	
SCT (um), mean ± SD	194.18 ± 5.68	217.29 ± 14.07	229.25 ± 13.89	*P* < 0.001^*,†,‡,§^
Choroid vascular parameters, mean ± SD				
TCA in mm^2^	0.81 ± 0.06	0.86 ± 0.09	0.90 ± 0.08	*P* < 0.001^*,†,‡^
LA in mm^2^	0.53 ± 0.05	0.54 ± 0.08	0.55 ± 0.08	*P* = 0.507^*^
SA in mm^2^	0.28 ± 0.03	0.31 ± 0.05	0.34 ± 0.03	*P* < 0.001^*,†,‡^
L/C ratio	0.65 ± 0.03	0.63 ± 0.05	0.61 ± 0.04	*P* = 0.019^*,†,‡,§^

DR, diabetic retinopathy; PRP, panretinal photocoagulation; NPDR, non-proliferative diabetic retinopathy; PDR, proliferative diabetic retinopathy; SCT, subfoveal choroidal thickness; SD, standard deviation; TCA, total choroidal area; LA, luminal area; SA, stromal area; L/C ratio, the ratio of the luminal to choroidal area

*: Statistical significance tested by ANOVA for normal distributions and Kruskal–Wallis tests for nonnormal distributions; all comparisons were corrected with the post hoc test

†: Significant difference between mean values of DM with no DR eyes and PRP-untreated NPDR eyes

‡: Significant difference between mean values of DM with no DR eyes and PRP-untreated PDR eyes

§: Significant difference between mean values of PRP-untreated NPDR eyes and PRP-untreated PDR eyes

The L/C ratio values in the three groups were 0.65 ± 0.03, 0.63 ± 0.05 and 0.61 ± 0.04, respectively. Relative to the eyes of DM with no DR, the L/C ratio values in eyes of PRP-untreated NPDR and PRP-untreated PDR patients were significant lower (*P* = 0.019), all three pairwise comparisons were statistically significant (Tukey-Kramer HSD, *P* < 0.05 for all comparisons). However, there was no significant difference in LA among the three groups (*P* = 0.507).

### Choroidal parameters between PRP-untreated and PRP-treated DR groups

Both NPDR and PDR eyes underwent OCT examinations after PRP treatment. The SCT values were lower in PRP-treated NPDR (214.94 ± 8.78um) than in PRP-untreated NPDR eyes (217.29 ± 14.07um), but not significantly. The SCT values were lower in PRP-treated PDR eyes (222.60 ± 11.62um) than in PRP-untreated PDR eyes (229.25 ± 13.89 um), but not significantly. There were no significant difference in TCA, LA, SA and the L/C ratio values in the groups. (Table 4 and Table 5).

**Table 4:** Choroidal parameters in PRP-untreated and PRP-treated NPDR eyes

	PRP-untreated NPDR	PRP-treated NPDR	P value
Number of eyes (n)	24	17	
SCT (um), mean ± SD	217.29 ± 14.07	214.94 ± 8.78	*P* = 0.068^*^
Choroid vascular parameters, mean ± SD			
TCA in mm^2^	0.86 ± 0.09	0.79 ± 0.12	*P* = 0.160^*^
LA in mm^2^	0.54 ± 0.08	0.50 ± 0.11	*P* = 0.394^*^
SA in mm^2^	0.31 ± 0.05	0.29 ± 0.06	*P* = 0.208^*^
L/C ratio	0.63 ± 0.05	0.63 ± 0.03	*P* = 0.204^*^

PRP, panretinal photocoagulation; PRP, panretinal photocoagulation; NPDR, non-proliferative diabetic retinopathy; SCT, subfoveal choroidal thickness; SD, standard deviation; TCA, total choroidal area; LA, luminal area; SA, stromal area; L/C ratio, the ratio of the luminal to choroidal area

*: Statistical significance tested by Independent t-test

**Table 5:** Choroidal parameters in PRP-untreated and PRP-treated PDR eyes

	PRP-untreated PDR	PRP-treated PDR	P value
Number of eyes (n)	20	20	
SCT (um), mean ± SD	229.25 ± 13.89	222.60 ± 11.62	*P* = 0.231^*^
Choroid vascular parameters, mean ± SD			
TCA in mm^2^	0.90 ± 0.08	0.83 ± 0.08	*P* = 0.448^*^
LA in mm^2^	0.55 ± 0.08	0.52 ± 0.07	*P* = 0.269^*^
SA in mm^2^	0.34 ± 0.03	0.32 ± 0.03	*P* = 0.073^*^
L/C ratio	0.61 ± 0.04	0.62 ± 0.03	*P* = 0.228^*^

PRP, panretinal photocoagulation; PDR, proliferative diabetic retinopathy; SCT, subfoveal choroidal thickness; SD, standard deviation; TCA, total choroidal area; LA, luminal area; SA, stromal area; L/C ratio, the ratio of the luminal to choroidal area

*: Statistical significance tested by Independent t-test

## Discussion

The microvascular complication-diabetic retinopathy (DR) is one of the most frequent complications of DM and affects the patient’s visual quality [15–17]. Since DR is one of the leading causes of blindness, the prevention and early detection of DR is key issue. With the development of EDI-OCT, researchers were able to accurately assess in vivo choroidal structure in a non-invasive way [18]. Since the choroid is responsible for supplying blood to the outer retinal layer, choroidal structure changes in DR patients might play an important role in the development of DR. [19] The relation between DM and SCT had been examined by some researchers in the recent years [20–22]. But different studies showed contradictory results. Querques et al. [8] showed patients with DM had significantly thinner choroids compared with normal controls. On the contrary, Kim et al. [21] showed that the healthy eyes had thinner choroids when compared to DM patients. Vujosevic et al. found no difference CT values between DM and normal controls [23]. In our study, we found that SCT was not significant different in 66 patients with DM as compared to controls (213.21 ± 19.02um vs. 212.63 ± 11.99, *P* = 0.849). But when we divided these DM patients into 3 groups (DM with no DR group, PRP-untreated NPDR group and the PRP-untreated PDR group), SCT were significantly lower in DM with no DR eyes compared with PRP-untreated NPDR and PRP-untreated PDR eyes. We suspected that SCT was reduced in the early stage of DM, and increased with progressive severity of DM. These findings carefully suggest that changes in choroidal vasculature could be the primary event in diabetes even where there is no DR.

Kim et al. reported that choroidal thickness increased significantly as DR worsened to PDR, and decreased in PRP-treated eyes. Choroidal blood flow increased in NPDR patients, and decreased in laser-treated PDR patients [24]. It has previously been reported that due to downregulation of vascular endothelial growth factor (VEGF), the choroidal blood flow is significantly reduced after PRP [25]. In this study, we also evaluated the effects of laser treatment on choroidal thickness and vascularity changes, we investigated eyes of 37 PRP-treated DR patients, including 17 NPDR eyes and 20 PDR eyes, separately. The mean SCT and the L/C ratio of the PRP-treated group did not differ significantly from that of the PRP-untreated group. There are several reasons for this result. First, the sample size may be too small. Second, choroidal measurements occur shortly after PRP treatment, which may not affect choroidal structural changes.

We can know from the above studies that the effect of DM on the choroid thickness changes remains unclear. There may be several reasons for this. First, the CT measurement process is different in these studies; second, the duration of DM can potentially affect the choroidal thickness; third, numerous physiologic variables can affect CT. All these reasons may lead to possible discrepancies in the results in different studies. Based on these factors, CT may not be a robust tool to evaluate the DR progression because there are many physiological factors such as diurnal variation, refractive error and age that affect it.

As the choroid is composed of blood vessels, connective tissue and extracellular fluid, measuring CT does not reflect which structure change within the choroid change. It is very meaningful to find a better indicator to quantify the changes of choroid structure. Several studies have been made to unfold the change of the typical angio-architecture of choroid in normal and diseased choroid [26–29]. Branchini et al. [30] first described the concept of analyzing choroidal vasculature in their study, they used customized software to calculate the ratio of light pixels to dark pixels in choroid. Sonoda et al. [11] used an image binarization tool to post-processing the OCT image and calculate the vascular value. Agrawal et al. [31] demonstrated relatively stable CVI to evaluate the choroid structure change, and because it is ratio defined as the proportion of LA to TCA, which is less affected by physiological factors.

Recently, there are more and more studies on the microscopic structure changes of diabetic retinopathy, and some researchers have focused on the relationship between CVI and choroidal microstructure changes in DR patients. Agrawal et al. [13] observed an increase in TCA in eyes of patients with DR. They hypothesized that as there is narrowing of capillaries in the choroid of the eyes of patients with DM and the proportion of vasculature-CVI would be decreased in patients with DM. Tan et al. [32] evaluated CVI in DM patients compared to controls and found that CVI was reduced (65.10 ± 0.20 versus 67.20 ± 0.16, *P* < 0.001), but they did not analyze CVI according to DR stage. In the study of Kim [14], they assessed choroidal changes in diabetic patients by measuring CVI and CT in conjunction with DR stage. These findings carefully suggest that changes in choroidal vasculature could be the primary event in diabetes even where there is no DR. CVI has also been widely used in other ocular diseases. Koh et al. [33] found CVI was lower in age-related macular degeneration (AMD) eyes as compared to normal controls, suggested that possible reduction in choroidal vascularity in eyes with AMD.

We used the L/C ratio to describe the change of choroid structure, which has the same meaning of CVI. In the study of Kim [14], they assessed choroidal changes in diabetic patients by measuring CVI and CT and found that the PDR eyes exhibited a significantly lower CVI value than the healthy control, DM with no DR, and NPDR eyes; the CVI in DM eyes was significantly lower than those of healthy controls even without DR. So they came to the conclusion that changes in choroidal vasculature could be the primary event in diabetes eyes even with no DR. Our research is different from their study, we compared LA, TCA and the L/C ratio in this study. We showed significantly smaller L/C ratio and bigger SA in patients with DM, compared to normal controls, regardless of the presence of DR. In this study, we also observed the choroidal structure indicators with different stages of DR, SA and TCA were significantly lower in DM with no DR eyes, followed by PRP-untreated NPDR eyes and the PRP-untreated PDR eyes (*P* < 0.001). The L/C ratio values were decreased in eyes of DM with no DR patients, PRP-untreated NPDR and PRP-untreated PDR patients (0.65 ± 0.03, 0.63 ± 0.05 and 0.61 ± 0.04, *P* = 0.019). But LA were not change with the progression of DR. This may because the thickening of the choroid in the different stages of DR is stromal thickening, not vascular change. Animal model showed that choroidal blood flow deficit can be an early pathologic change in DR [34]. Similar to animal research, with the progression of DR, the diameter of choroidal vessels may reduce due to vascular constriction secondary to choroidal hypoxia, and changes of choroidal blood flow may occur before retinopathy manifestation [35, 36]. Choroidal blood flow deficit can be an early pathologic change in DR, as shown in an animal model. These findings carefully suggest that changes in choroidal vasculature could be the primary event in diabetes even where there is no DR.

The current research investigated the CT and L/C ratio and their correlation with severity of DR in DM patients. Our study selected new parameters that represent the microstructure of the fundus and found SCT increased, but the L/C ratio values were significantly decreased with severity of DR eyes compared with DM and normal eyes. However, our research also has some limitations. Firstly, the sample size of this study was relatively small, which may have limited the statistical strength of the analysis and reduced our ability to perform correlational analyses for DM and the fundus microstructure. Future studies should be performed with larger cohorts and longer follow-up periods to determine the fundus microstructure changes in patients with different degrees of DR. Secondly, several compounding factors, such as diurnal variation, age, sex, refractive error, systolic BP, axial length, anterior chamber depth, and lens thickness were not considered in this study. Thirdly, because the SCT of diabetic macular edema (DME) eyes differ from that of normal eyes, we did not included DME eyes in the study, which was another limitation. The forth limitation was that our image processing technology clearly displays and quantifies vascular tissue in the choroidal cross section, but it can only reflect the choroidal vasculature change in a certain part, not a wide range of fundus choroidal change. Therefore, future research can analyze choroidal vascular density in a wide range of fundus with optical coherence tomography angiography (OCTA) simultaneously. Later studies require OCTA devices with better choroidal imaging quality to analyze choroidal vascular changes.

## Conclusions

In conclusion, SCT increased, but the L/C ratio significantly decreased with severity of DR eyes compared with DM eyes and normal. Choroidal blood flow deficit can be an early pathologic change in DR. The L/C ratio may predict DR development before they are otherwise evident clinically. Ischemic changes in choroidal vasculature is the primary event in diabetes, even when DR is absent.

## Additional files

Additional file 1: Demographic and clinical data in this study. (XLSX 24 kb)

Additional file 2: OCT data in this study. (XLSX 38 kb)

## Data Availability

A supplemental material which included the primary data has been uploaded accordingly (see Additional files 1 and 2).

## Abbreviations

BCVA: Best-corrected visual acuity
BMI: Body mass index
DM: Diabetes mellitus
DME: Diabetic macular edema
DR: Diabetic retinopathy
EDI-OCT: Enhanced depth imaging optic coherence tomography
FA: Fluorescence angiography
IOP: Intraocular pressure
L/A ratio: L/C ratio
LA: Luminal areas
OCTA: Optical coherence tomography angiography
PRP: Panretinal photocoagulation
RNFL: Retinal nerve fiber layer
SA: Stromal area
SCT: Subfoveal choroidal thickness
TCA: Total choroidal area
